# Chromosome-level genome assembly of trypanosomatid parasite *Lotmaria passim* links chromosome duplication and divergence with infection of honey bees

**DOI:** 10.1186/s12864-025-12082-y

**Published:** 2025-12-01

**Authors:** Anthony Nearman, Anzhelika Butenko, Jay D Evans, Evan C Palmer-Young

**Affiliations:** 1 USDA-ARS Bee Research Laboratory, 10300 Baltimore Ave, BARC-East Bldg 306 Rm 313, Beltsville, MD 20705 USA; 2 Czech Academy of Sciences, Institute of Parasitology, České Budějovice, 370 05 Czech Republic; 3 Life Science Research Centre, Faculty of Science, University of Ostrava, Ostrava, 710 00 Czech Republic; 4 Faculty of Science, University of South Bohemia, České Budějovice, 370 05 Czech Republic

**Keywords:** Aneuploidy, Trypanosomatid ancestral supernumerary chromosome, Gene duplication, Phylogenomics, Polycistronic genome organization, Post-transcriptional gene regulation, Differential gene expression, Host-parasite interactions, *Crithidia*

## Abstract

**Background:**

The protist family Trypanosomatidae includes parasites of insects, vertebrates, plants, and even other unicellular eukaryotes. The genomes of these species harbor clues to the evolution of parasitism, adaptation to new hosts, and infection of mammals. We present an analysis of a chromosome-level genome assembly of *Lotmaria passim*, the most prevalent known trypanosomatid of honey bees, linking genome sequence and organization to gene expression and infection of bees.

**Results:**

The genome showed a high degree of synteny with assemblies of other trypanosomatids and especially to the closely related *Leptomonas pyrrhocoris*. It included four copies of chromosomes that shared ancestry with the tetrasomic *Leishmania* Chromosome 31 and are consistently supernumerary throughout Trypanosomatidae. However, these chromosomes showed lower similarity to *L. passim* relatives than did the genome overall, with sufficient variation across haplotypes to distinguish two separate disomic chromosomes. Transcriptomic analyses showed that these chromosomes are enriched in genes upregulated during bee infection, and each include five paralogs of the GP63 gene implicated in infection of both insects and mammals. Patterns of expression in bees suggested decreased protein synthesis, a shift from carbohydrate- to amino acid-based metabolism, and reduced cell motility in bee guts versus cell culture. In contrast, genes involved in cell adhesion were upregulated, consistent with the importance of attachment to insect tissue in this species and the family overall.

**Conclusions:**

Our analysis links differentiation of a conserved supernumerary chromosome with infection of bees, parallel to this chromosome’s role in *Leishmania* infection of mammals and linking chromosome-level changes with adaptation to new hosts.

**Supplementary Information:**

The online version contains supplementary material available at 10.1186/s12864-025-12082-y.

## Introduction

The trypanosomatids are an intriguing and medically important family of protists that exploit a variety of ecological niches. They include obligate monoxenous (single-host) parasites of insects and dixenous (two-host) insect-vectored parasites of plants and vertebrates [1–3], namely the *Leishmania* and *Trypanosoma* species that cause considerable neglected diseases in tropical and subtropical regions [4–6]. The factors that enable the radiation of trypanosomatids into novel host environments are therefore of basic and applied interest [2, 7].

The sequencing of genomes from trypanosomatids across this spectrum of lifestyles has begun to reveal the phylogenetic relationships between species, changes associated with the transition to parasitism, and adaptations to different niches [2, 8–10]. Several genetic features have emerged from the species investigated thus far. These include a generally conserved genome structure with high degree of synteny among distantly related species, the arrangement and transcription of genes in long polycistronic blocks, and predominantly post-transcriptional regulation of gene expression [11, 12].

Many species also exhibit duplication of chromosome segments and variation in chromosome copy number, or aneusomy [9, 13, 14]. Although such duplications create a potentially costly excess of the genes present in the supernumerary region, they can also benefit fitness through increased dosage of specific advantageous genes [14]. This can facilitate adaptation to ecological and evolutionary pressures, such as exposure to toxins and new host environments [14–17]. One remarkable example is the *Leishmania* Chromosome 31, which is consistently tetrasomic (i.e., present as four copies) across species and strains in this genus [14, 17]. It is also enriched in genes that are upregulated during infection of vertebrates– including those encoding amastins as well as amino acid transporters– suggesting that this duplication aided the ability to colonize these new hosts [16]. A recent investigation of over 800 trypanosomatid genome assemblies revealed that scaffolds and chromosomes syntenic with Chromosome 31 are in fact tetrasomic across a wide range of mono- and dixenous species, suggesting that this aneusomy is ancestral in the Trypanosomatidae family rather than a specific adaptation of *Leishmania* [14]. However, its importance for the infection of other hosts and insect vectors remains unclear.

Among the monoxenous trypanosomatids, several species are associated with bees [18]. *Crithidia bombi* and *C. expoeki*, the main species identified in bumble bees, have emerged as models of host-parasite ecology and evolution and revealed the context- and life stage-dependent effects of trypanosomatid infection on insects [19–21]. *Lotmaria passim*, on the other hand, is the most prevalent trypanosomatid species known in honey bees [18]. The parasite colonizes the walls of the honey bee ileum and rectum [18, 22], with fecal-oral transmission inferred from its presence in honey bee feces [23]. It exhibits a global distribution, high prevalence, negative effects on bee survival, and an association with collapsing colonies [24–33], suggesting its importance as a threat to bee health. In addition, *L. passim*’s bee gut niche offers several parallels with the intracellular niche of *Leishmania* in vertebrates [2, 34]. These include exposure within bees to elevated temperatures similar to those of the mammalian bloodstream [35, 36]; and establishment in the acidic environment of the bee hindgut [37], where pH resembles that in the acidic phagolysosome colonized by *Leishmania* amastigotes [38, 39]. Hence, its adaptations to this niche could provide insights into trypanosomatids’ infection of mammals.

Our group recently published a high-quality, chromosome-level assembly of the BRL (Bee Research Lab) type strain (American Type Culture Collection isolate PRA-422, GCA_037349495.1) of *L. passim* [40]. The culture was isolated from the ileum of adult female *Apis mellifera* at the United States Department of Agriculture Bee Research Lab in Beltsville, Maryland, USA in September 2012 [18]. Compared to the draft genome of strain SF (San Francisco, ATCC isolate PRA-403) published ten years prior—before the differentiation of *L. passim* from its relative *C. mellificae* [41] — the new assembly is more contiguous (number of nuclear genome scaffolds decreased from 2801 to 31), complete (the representation of Euglenozoa BUSCO genes increased from 90 to 98.5%), contains mitochondrial genome sequences, and enabled discernment of chromosome-level aneusomy [40]. To our knowledge, it represents the first chromosome-level assembly for a species in the Crithidiatae clade comprised of *Lotmaria*,* Leptomonas*, and *Crithidia* [8]; the first for a monoxenous species in the Leishmaniianae subfamily; and the second for a monoxenous trypanosomatid (after *Angomonas deanei* [42]). This yielded a more complete understanding of the genome’s structure, while providing a framework to investigate the relationship between gene arrangement, expression, and function. One of the striking elements of the assembly was the presence of a paralogous chromosome pair represented by Chromosomes 5 and 6, the origins and significance of which remained unclear [40].

To discover the distinguishing features of the *L. passim* genome–including its paralogous chromosome pair–and their relevance to infection of honey bees, we compared the structure of the genome with that of related trypanosomatids, quantified spatial patterns of gene expression within and across chromosomes, and analyzed functional changes in gene expression of parasites in bee guts relative to cell cultures. Our results highlight the evolution of *L. passim*’s two paralogous chromosomes and their orthology with *Leishmania* spp. supernumerary Chromosome 31. We also link genes on these chromosomes with infection of bees and point to the importance of attachment to host tissue in the parasite’s life cycle.

## Methods

### Synteny analysis

The genome of the *L. passim* BRL type strain (maintained in the American Type Culture Collection as isolate “PRA-422”, GenBank assembly GCA_037349495.1) was previously sequenced using a combination of Pac-Bio HiFi and Illumina Hi-C technologies, assembled to 31 nuclear chromosomes, and annotated with 10,288 genes [40].

Conservation of gene order (synteny) between *L. passim* and other Leishmaniianae species with high-quality assemblies (*Leptomonas seymouri*; *Crithidia bombi*, *C. expoeki*, and *C. fasciculata*; *Leishmania major* and *L. braziliensis* (Supplementary Table S1)), including Crithidiatae genomes with < 250 scaffolds and one chromosome-level assembly from each subgenus of *Leishmania*, was quantified with SyMAP v5.9.9 [43] using default parameters. SyMAP raw data was exported and visualized using the R package circlize v0.4.16 [44].

To examine telomeric sequences, tandem repeats found at chromosome termini were identified and counted using the Telomere Identification toolKit [45]. We used the ‘explore’ function to identify repeated sequences from 5 to 15 bp long; and the ‘search’ function to detect matches to the canonical sequence “CCCTAA”, used as a marker for trypanosomatid telomeres [46], within 1% of each chromosome’s length from either end.

### Phylogenetic reconstruction

To define the phylogenetic relationships between *L. passim* and other trypanosomatids, we constructed a tree based on predicted proteins from 15 *Lotmaria*,* Leptomonas*,* Crithidia*, and *Leishmania* species in the Leishmaniianae subfamily (Supplementary Table S2). For species without publicly available annotations, we used those made in a previous phylogenomic analysis [8]. Proteins were aligned using MAFFT through Orthofinder v3.0.1b1, resulting in 2,530 single-copy orthologs from which a phylogenetic tree was inferred using fasttree [47]. Orthofinder was run with default parameters except -M msa to enable multisequence alignment and tree inference. Tree visualization was performed using R packages ape v5.8.1 and ggtree v3.16.3 [48, 49].

### Spatial analysis of genes and gene expression

To quantify the spatial clustering of genes by strand within each chromosome, we created a matrix for all gene pairs and their distances apart along the chromosome. We used this to model the correlation between log_10_(pairwise distance) (included as both a linear and quadratic term) and the probability of genes being on the same strand using a binomial model with chromosome as a random effect. This and subsequent models were implemented in R version 4.4 using package glmmTMB v1.1.12 to construct models, car v3.1.3 to evaluate significance of predictor terms, emmeans v1.11.2.8 to compute marginal means for different values of predictor variables, and ggplot2 v3.5.2 to visualize results [50–53]. We also used a binomial model to compare the proportion of features annotated as pseudogenes on the paralogous Chromosomes 5 and 6 vs. the rest of the genome.

We then combined our new genome annotation with publicly available transcriptomic data to examine chromosome-scale patterns of gene expression and up- and downregulation in honey bee hindguts relative to parasite cell cultures. The transcriptomic data (originally described in [32]) consisted of 30 samples from the type strain ‘SF’ (ATCC isolate ‘PRA-403’ [41]) grown in antibiotic-supplemented ‘FPFB’ medium [54] and gut samples of parasite-inoculated honey bees (*Apis mellifera*) at 7, 12, 20, and 27 d post-inoculation (*n* = 6 replicate libraries per group). Reads from the associated SRA files were mapped to the *Lotmaria passim* genome (GCA_037349495.1) using the short read alignment software STAR v2.7.11b [55] using default alignment parameters for unpaired reads and summarized by gene using featureCounts v2.0.6 [56]. Reads that mapped to multiple genes were fractionally distributed among each potential gene from which they could have originated (featureCounts -M --fraction). One of the replicates for the 7 d time point was excluded due to low read counts. Differential expression was analyzed using R package DESeq2 v1.48.2 [57] to compare expression in the bee gut relative to cell culture at each time point post-inoculation (see Supplementary Methods for additional details). Differences in proportions of differentially expressed genes between the paralogous chromosomes and the rest of the nuclear genome were evaluated using binomial models for five different differential expression categories: Statistically significant upregulation, downregulation, or differential expression in either direction with respect to cultured cells (adjusted p-value < 0.05, any fold change); and strong (adjusted p-value < 0.05 and |fold-change| >2) up- and downregulation. Because the gene expression patterns within each chromosome were generally consistent across the four time points, we fit a single model for each expression category with chromosome type (Chromosomes 5 and 6 vs. rest of genome) as a fixed predictor and time point as a random effect. We also compared the absolute expression level (measured as log_2_(transcripts per million (TPM)), pooled across all libraries) between genes on Chromosomes 5 and 6 vs. other chromosomes.

The amount of variance in expression (i.e., gene length-corrected read count) explained by same-stranded gene clusters of more than 10 consecutive genes was tested using linear mixed models. We fit separate models for each of the five treatment groups, then extracted the proportion of variance explained by the gene cluster random effect term (see Supplementary Methods). We also analyzed correlations in absolute and differential expression between adjacent genes on the same vs. opposite strand of the chromosome (see Supplementary Methods).

### Functional gene annotation and patterns of expression

We conducted functional analyses of differential gene expression in the bee gut vs. cell culture using Gene Set Enrichment Analysis (GSEA [58]) of Gene Ontology (GO [59, 60]) and Kyoto Encyclopedia of Genes and Genomes (KEGG [61]) terms. Genes were assigned tentative GO terms by submitting the annotated protein sequences to the PANNZER2 server [62]. Terms were filtered to retain only predictions with a positive prediction value of > 0.5, resulting in annotations for 2,457 of the original 10,288 submitted sequences. Parent terms in the GO hierarchy implied by each prediction were added using the buildGOmap function of R package ClusterProfiler v3.21 [63]. The resulting set of terms was then filtered using FunTaxIS-lite to remove terms unlikely to occur in trypanosomatids [64]. Annotated genes were similarly assigned KEGG functions based on protein sequences and annotated to associated pathways using BlastKOALA [65], which inferred KEGG terms for 2,693 of the submitted protein sequences. The resulting list of terms was filtered to retain only pathways annotated in reference kinetoplastid genomes present in the KEGG database.

Gene set enrichment analysis, which uses a rank-based enrichment score to identify gene sets whose genes are overrepresented at the top and bottom of a ranked gene list [58], was implemented on gene sets associated with each GO term or KEGG pathway using ClusterProfiler [63] on all terms associated with at least 15 genes. Genes were ranked within each time point using the test statistic from the differential expression estimate for bee gut-derived relative to cultured cells (i.e., the ratio of the estimated log2-fold change to the standard error of the estimate) as the signal-to-noise ratio (details in Supplementary Methods). We discuss only gene sets with adjusted p-values < 0.01.

## Results and discussion

### Synteny with divergence of the ancestrally tetrasomic chromosome

**Fig. 1:**
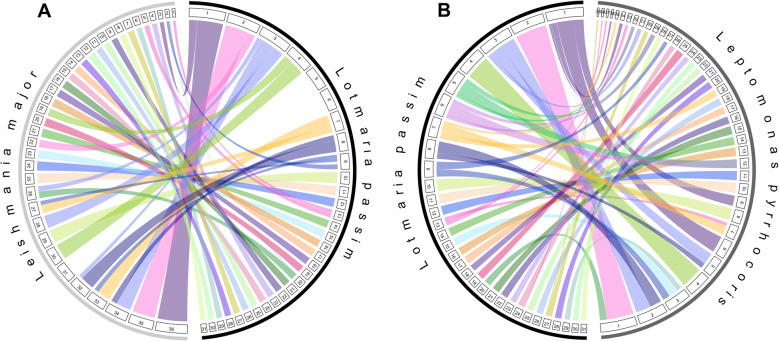
Synteny of* L. passim* chromosomes with trypanosomatid relatives *Leishmania major* and *Leptomonas pyrrhocoris.* Labeled bands depict scaffolds of *L. passim* and (**A**) *L. major* or (**B**) *L. pyrrhocoris*. Arcs connect syntenic regions. **A** The paralogous *L. passim* Chromosomes 5 and 6 and the tetrasomic *L. major* Chromosome 31 constitute the only chromosome-scale synteny gaps. **B** All major genomic regions are shared between *L. passim* and *L. pyrrhocoris*, with the region syntenic with paralogous Chromosomes 5 and 6 split across 3 *L. pyrrhocoris* scaffolds

**Fig. 2:**
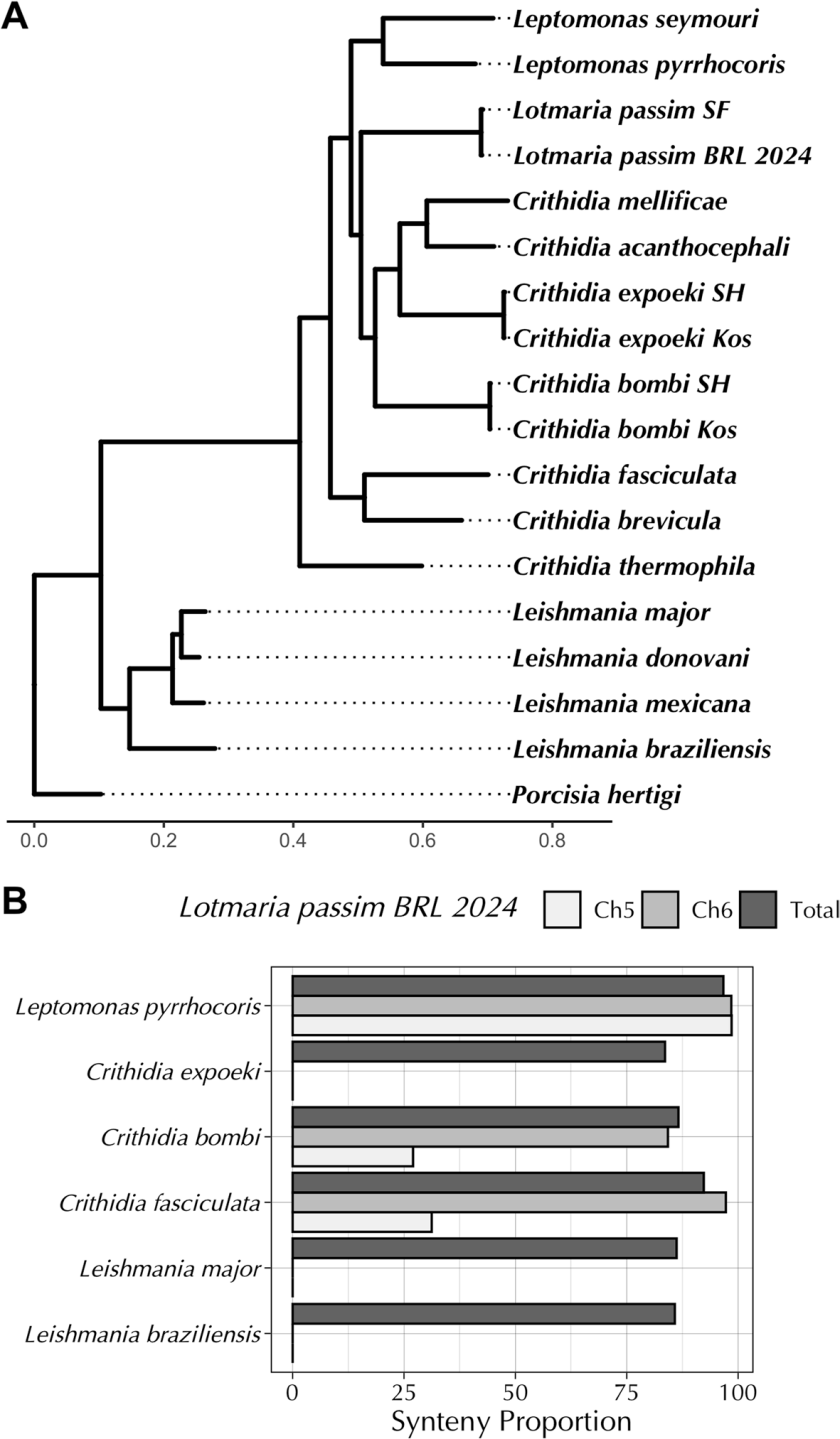
Phylogenetic placement and proportions of the *L. passim* genome and paralogous Chromosomes 5 and 6 syntenic with genomes of selected relatives. **A** Phylogenomic tree depicting relationships between *L. passim*,* L. pyrrhocoris*, and other trypanosomatids in the Leishmaniianae subfamily. The tree was inferred from protein sequences of 2530 single-copy orthologs. Our assembly is labeled ‘BRL (2024)’. The position of the ‘SF’ strain used for the first draft genome assembly is also shown [41]. For *C. bombi* and *C. expoeki*, nodes are shown for both the original published annotations by Schmid-Hempel and colleagues (‘SH’ [89]) and independent annotations of the same assemblies created in a review of trypanosomatid phylogenomics by Kostygov and colleagues (‘Kos’ [8]). All nodes had support values of 1 based on 1,000 resamples. **B** Synteny of paralogous chromosomes and genome overall. Y-axis shows species analyzed; X-axis represents proportion of the *L. passim* genomic of chromosome region covered by synteny blocks. Shading of bars corresponds to the region quantified. “Total” indicates the entire nuclear genome

The *L. passim* nuclear genome was assembled to 31 chromosome-level scaffolds, along with the mitochondrial (kinetoplast) maxi- and minicircles typical of trypanosomatids, as described previously [40]. The genome assembly displayed a high degree of synteny with related species in the Leishmaniinae subfamily (Fig. 1), supporting the phylogenetic placement of *L. passim* within the *Crithidia/Leptomonas/Lotmaria* clade (Fig. 2A). Synteny was highest with the close relative *Leptomonas pyrrhocoris*, for which the *L. passim* genome was 97% covered by 49 synteny blocks, including 62 sets of at least 20 collinear genes (Figs. 1 and 2B). However, synteny was also strong with *Crithidia bombi* (87% across 71 blocks) and *C. fasciculata* (92% across 57 blocks), and slightly lower but with fewer blocks for the chromosome-level assemblies of *Leishmania major* (86%, 41 blocks) and *L. braziliensis* (86%, 48 blocks) (Fig. 2B). In four cases (Chromosomes 1, 2, 4, and 13), *L. passim* chromosomes displayed synteny to multiple full chromosomes of *L. major* (Fig. 1A). For example, *L. passim* Chromosome 1 incorporated synteny blocks spanning *L. major* Chromosomes 36 and 3, similar to the fusion of *L. major* Chromosomes 36 and 20 in *L. mexicana* [16].

**Fig. 3:**
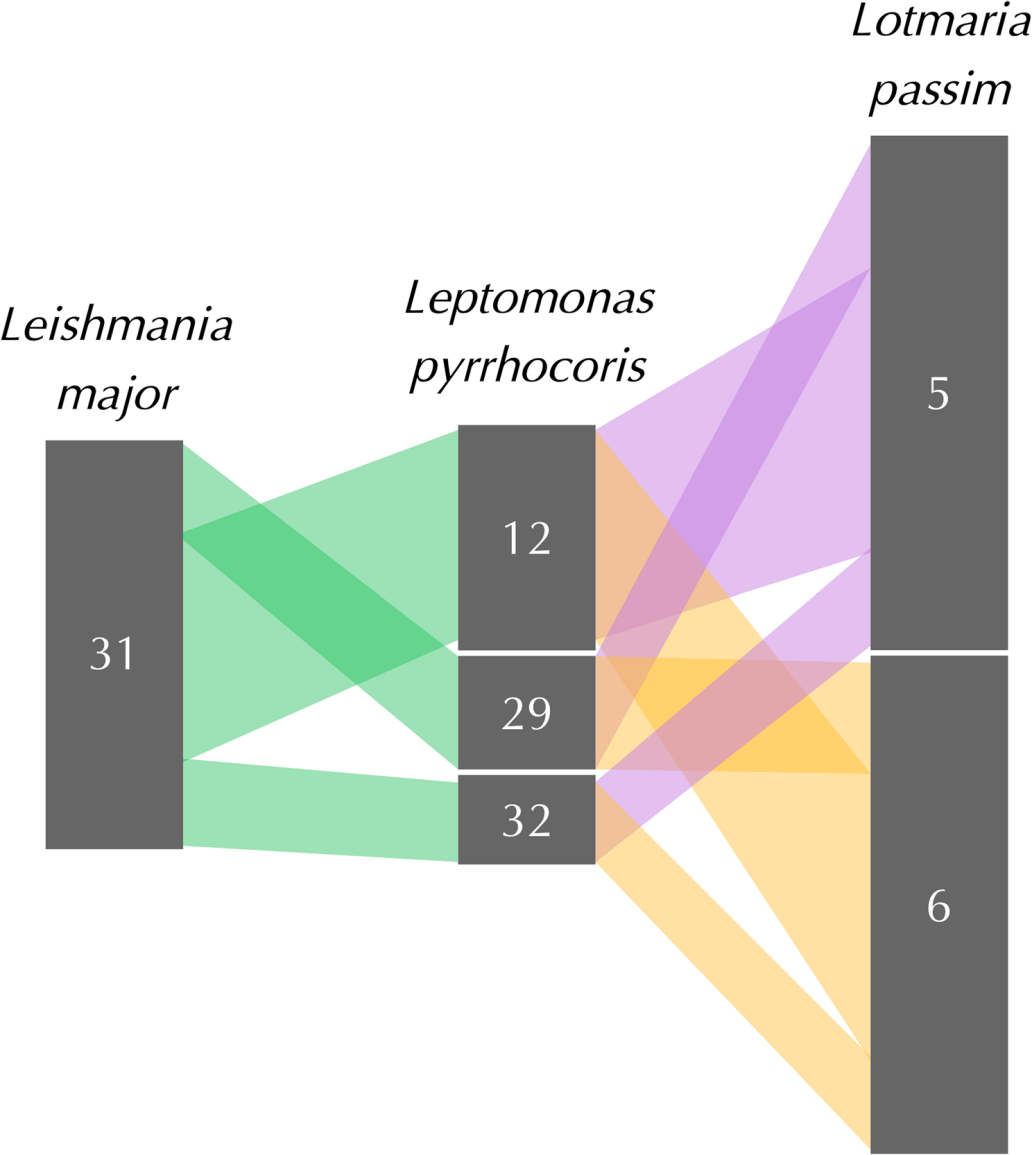
Three-way synteny between *L. passim* Chromosomes 5 and 6; *L. pyrrhocoris* tetrasomic scaffolds 12, 29, and 32; and *L. major* tetrasomic Chromosome 31. The *L. passim* Chromosomes 5 and 6 are syntenic with the same *L. pyrrhocoris* scaffolds that showed synteny with *L. major* Chromosome 31. This strongly suggests shared ancestry between the corresponding chromosomes of *L. passim* and *L. major*

Compared to the rest of the genome, the paralogous *L. passim* Chromosomes 5 and 6 showed substantially lower levels of synteny with homologous chromosomes in most of the trypanosomatid relatives we examined, with the exception of *L. pyrrhocoris* (Fig. 2B). Only around half the combined length of these chromosomes showed synteny with *C. bombi* and *C. fasciculata*, and no synteny was detected with *C. expoeki* or either species of *Leishmania*, contrasting with the > 80% synteny of the genome overall (of which Chromosomes 5 and 6 constitute ~ 12%) for each of these comparator species (Fig. 2B). However, each of Chromosomes 5 and 6 was syntenic with the tetrasomic scaffolds 12, 29, and 32 of the *L. pyrrhocoris* assembly (Fig. 3) [9, 14], suggesting that these three scaffolds (ordered 32, 12, 29) comprise a single *L. pyrrhocoris* chromosome. These *L. pyrrhocoris* scaffolds were in turn syntenic with the ancestrally tetrasomic Chromosome 31 of *L. major*, strongly suggesting that these *L. passim* chromosomes share ancestry with the *Leishmania* Chromosome 31. This synteny, however, was not inferred when comparing *L. passim* with *L. major* directly (Fig. 1A). These findings suggest that Chromosomes 5 and 6 have substantially diverged from their ancestral state in *L. passim* and compared to the equivalent genomic regions of other monoxenous species.

**Fig. 4:**
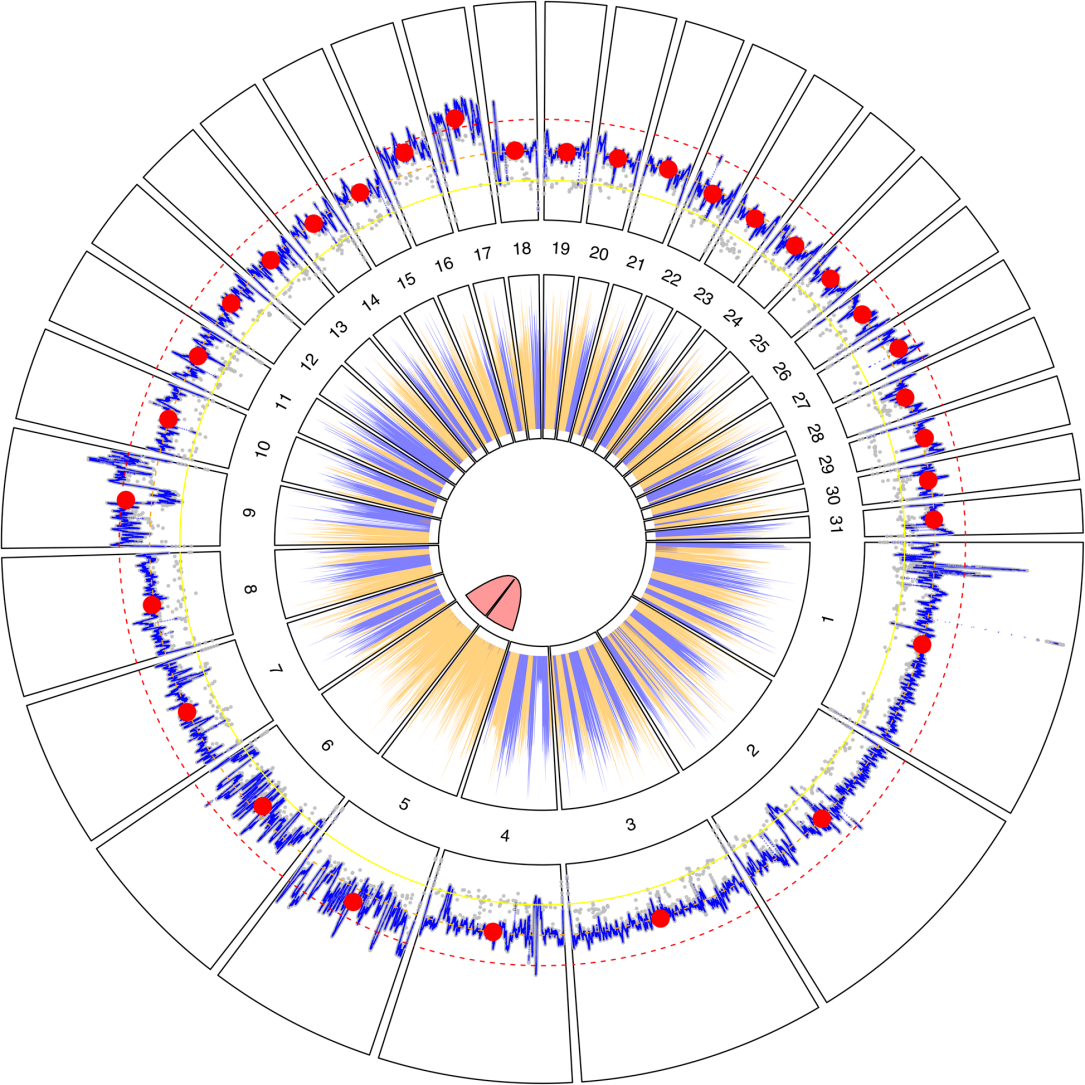
Circular representation of *Lotmaria passim* BRL (2024) genome assembly. Radial segments correspond to the 31 nuclear chromosomes, numbered by size from largest to smallest. Outer ring: sequencing depth. Gray points show base-level read depth, subsampled at 100 bp intervals. Blue line trace represents 1 Kb moving average. Large red circle represents the chromosome-level median. Faint scatter of gray points with roughly half the read depth of the chromosome overall suggests heterozygous sites. Concentric yellow, orange, and red lines represent 50%, 100%, and 150% of the median chromosome-level depth; this corresponds to expected depths for monosomic, disomic, and trisomic chromosomes, respectively. The paralogous Chromosomes 5 and 6 appear as disomic chromosomes with distinct coverage traces. Inner ring: relative density of coding regions (percent of each 10 Kb interval covered by predicted exons), colored by strand (blue: positive, orange: negative), suggesting long stretches of polycistronic genes on the same strand and strand-biased gene arrangement on Chromosomes 5 and 6. Opaque red link between Chromosomes 5 and 6 indicates the similarity between these two regions

The combination of a high quality assembly and high nucleotide divergence between the two haplotype pairs (88.8% sequence identity) allowed us to distinguish two separate disomic chromosomes in *L. passim* (Fig. 4). Our finding was supported by both median sequencing depth–which was within 20% of the overall chromosome-level median for each of the two scaffolds–and variant calling, which was consistent with a 50:50 distribution of variants at polymorphic loci [40]. Although this contrasts with the tetrasomy concluded for the other species [14, 16], divergence of these chromosomes is consistent with the high nucleotide diversity of the tetrasomic genome scaffolds within populations of *Crithidia*,* Leptomonas*, and *Leishmania* relatives [14]. Additional haplotype-level sequences of this tetrasomic region in other trypanosomatids are needed to determine whether its assortment into two chromosome pairs is unique to *L. passim*. However, analysis of the *L. major* Friedlin genome found evidence for only 239 polymorphic sites on Chromosome 31 [66], suggesting > 99.98% identity across haplotypes over this ~ 1.5 Mb region.

The validity of the assembled chromosomes was supported by the presence of telomeric repeats at their termini. Most of the telomeres contained the canonical tandem arrays of “CCCTAA” commonly used to distinguish trypanosomatid telomeres [46] (Supplementary Fig. 1). However, other tandem repeats that did not include this motif were found as well, accounting for 45% of the total number (Supplementary Table S3). For example, Chromosome 7 began with over only 2 repeats of “CCCTAA” (Supplementary Fig. 1) followed by 3 Kb of repeating “ACACCCGT”.

Although slight variations in CCCTAA-containing repeats have been found across species and among chromosomes of *Leishmania* species [67, 68], our assembly suggests a diversity of repeat structures that has not been resolved by the use of CCCTAA-based probes [46], and will require additional chromosome-level assemblies to better understand.

### Structural patterns of gene arrangement and transcription

#### Genes are arranged in polycistronic clusters

Our annotation of genes indicated the presence of long clusters of genes on the same strand that in some cases spanned entire chromosomes (Fig. 4, inner ring shading), consistent with the previously described organization of trypanosomatid genes in long polycistronic units [11]. Adjacent gene pairs had a 97% chance of being located on the same strand; genes within 1 Kb of one another had a > 90% chance of sharing a strand, and those within 10Kb a > 80% chance (Supplementary Fig. 2). The strand-wise arrangement of genes was conspicuously strong on the paralogous Chromosomes 5 and 6 (Fig. 4, inner ring shading), where essentially all (96%) of the annotated regions (including intact protein-coding genes as well as non-coding RNAs and predicted pseudogenes) were on the negative strand, consistent with annotations of the tetrasomic chromosomes with shared ancestry in other trypanosomatids [14, 16].

#### Ancestrally tetrasomic, paralogous Chromosomes 5 and 6 are enriched in genes upregulated during infection

**Fig. 5:**
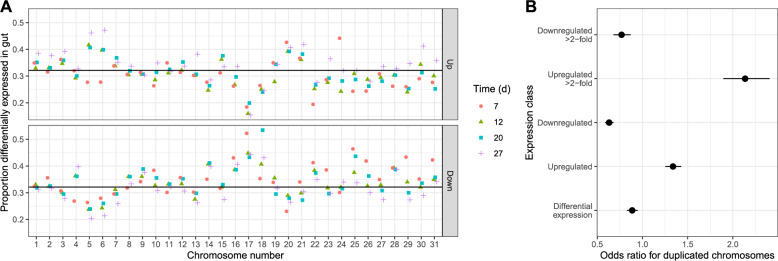
Paralogous Chromosomes 5 and 6 are enriched in genes upregulated during infection of honey bees. **A** Proportions of genes significantly (*P* < 0.05) up- and down-regulated for each chromosome and time point post-inoculation. Horizontal lines indicate median proportions. Shapes and colors represent the four times of sampling, in days post-inoculation. **B** Exponentiated model coefficients for proportions of genes significantly (*P* < 0.05) and strongly (*P* < 0.05 and > 2-fold change) up- and down-regulated in bee gut vs. culture environments. X-axis shows exponentiated coefficients and 95% confidence intervals for genes on Chromosomes 5 and 6 versus the rest of the genome

We used publicly available *L. passim* transcriptomic data [32] in combination with our new chromosome-level genome assembly and its annotation to re-examine structural and functional patterns of gene expression during infection of honey bees. Roughly two-thirds of annotated genes were differentially expressed in bees vs. log-phase cell cultures at each of the four time points post-inoculation (Fig. 5A). The paralogous Chromosomes 5 and 6 were conspicuously enriched in genes upregulated in the bee gut environment, with higher probabilities of genes being upregulated and lower probabilities of genes being downregulated (Fig. 5A). Across all time points, genes on these two chromosomes were more than twice as likely to be upregulated by greater than two-fold relative to genes on other chromosomes (Fig. 5B). However, genes on the duplicated chromosomes were expressed at more than two-fold lower levels (i.e., fewer read counts) than those on other chromosomes (Supplementary Fig. 3; Supplementary Fig. 4), thereby compensating for the doubling of copy numbers resulting from chromosome duplication. A similar pattern, in which genes on the ancestrally tetrasomic chromosome are collectively expressed at similar levels to those on disomic chromosomes despite having twice as many copies, was likewise reported for the *Leishmania* Chromosome 31 [14]. The paralogous chromosomes were also enriched in predicted pseudogenes, with a > 50% increase in proportions of this feature type relative to the rest of the genome (Supplementary Fig. 5).

#### Transcript abundances show traces of polycistronic transcription

**Fig. 6:**
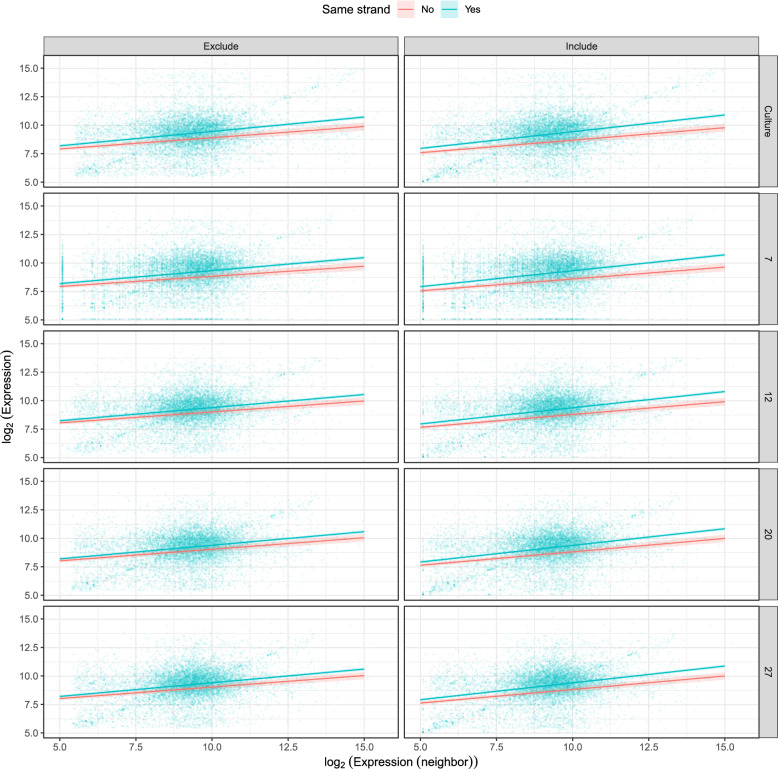
Expression levels of adjacent genes are correlated, with stronger correlations between neighboring genes located on the same strand of the chromosome. Y- and X-axes show log-transformed expression levels (transcripts per million) of each pair of neighboring genes. Trendlines show correlations with 95% CI shaded bands, in red for neighbors on opposite strands and blue for neighbors on the same strand. Panels in rows indicate sample groups (cultured cells and cells from bee guts at different time points post-inoculation). Panels in columns show data with and without low-count (i.e., low expression level) genes excluded

We quantified spatial patterns of gene expression within chromosomes and their relationship to strand-wise gene arrays, which are canonically transcribed as a single polycistronic unit [11]. We found weak (correlation coefficient 19–24%) but highly significant positive correlations between expression levels of adjacent genes in each sample group (e.g., ꭕ^2^_1_= 671, P < 0.001 for cell cultures with low-count genes excluded). However, the correlation between neighbors was stronger for neighboring genes on the same versus opposite strands of the chromosome, as evidenced by the significance of the neighbor expression x shared strand interaction term (e.g., ꭕ^2^_1_= 23.6, P < 0.001 for cell cultures, Fig. 6). The fixed-effects model explained 5–10% of the variance in each sample group, suggesting a detectable signal of polycistronic transcription despite considerable post-transcriptional regulation of mRNA levels; this observation might be at least partly attributed to the origin of a portion of sequencing reads from precursor mRNAs. Same-stranded arrays of > 10 consecutive genes explained 8–16% of variation in expression levels when modeled as a random effect, depending on whether genes with low read counts were excluded (Supplementary Fig. 6).

We also found positive correlations between differential expression of neighboring genes in bee guts vs. cell culture, although these were less pronounced than for absolute expression, and no significant difference was evident between gene pairs on the same vs. opposing strands (i.e., P > 0.05 for neighbor log_2_-fold change by shared strand interaction term, Supplementary Fig. 7, Supplementary Table S4). The proportion of variance explained by the fixed effects model (comprising log_2_-fold change of the neighboring gene, whether neighbors were on the same strand, and their interaction) was < 1% for every time point, indicating that differential expression of neighboring genes is only weakly interdependent.

Several factors could contribute to the relatively low correlations in transcription between genes within polycistronic gene clusters in our analysis. Technically, it is difficult to define true transcription start sites. In trypanosomatids, such sites lack promoter motifs and can include multiple potential start positions that span a 50–100 bp region epigenetically marked by histone acetylation [69]. In the absence of epigenetic evidence, our gene clusters were delineated based on the strand switches of genes in our annotation and may not correspond to the actual transcriptional units. Trypanosomatid mRNA abundance is subject to extensive post-transcriptional regulation, most notably by RNA-binding proteins that mainly bind to 3’ untranslated regions and modulate mRNA stability [11]; this compensates for the lack of gene-level transcriptional control. Indeed, the fact that some of our sequencing reads likely originate from precursor (rather than exclusively mature) mRNAs not yet subjected to these regulatory mechanisms could have resulted in generous estimates of intergenic correlation in our analyses.

### Functional analysis of transcription

We used the annotation of our new genome assembly [40], which identified roughly 2,000 more protein-coding genes than the annotation of the draft genome assembly [41] used previously for RNAseq analysis of *L. passim* [32], to analyze functional patterns of differential gene expression in the gut vs. cell culture environment using Gene Set Enrichment Analyses. This method, which characterizes the rank-based distribution of changes in expression level for the set of genes associated with each function-related term [58], offers a more quantitatively nuanced alternative to the binomial enrichment tests for overrepresentation used by the dataset’s original authors [32], incorporating information about the magnitude of differential expression rather than relying on arbitrary distinctions between genes that are differentially expressed or not. We also conducted a pathway analysis using the Kyoto Encyclopedia of Genes and Genomes (KEGG) database [61], which contains detailed information on metabolic and other pathways, including those specifically related to trypanosomatid infection.

#### Upregulation of peptidase activity and cell adhesion; downregulation of carbohydrate metabolism, protein synthesis, and cell motility during infection

**Fig. 7:**
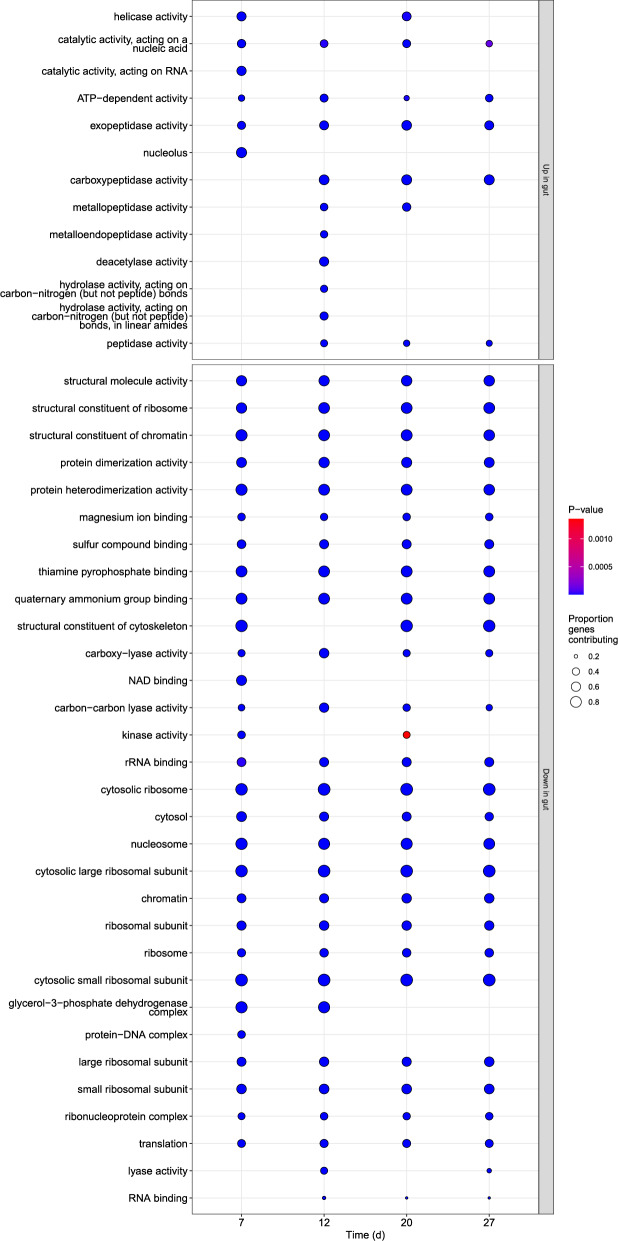
Gene Ontology gene set enrichment for *L. passim* expression levels in honey bee gut samples at 4 time points post-inoculation relative to cultured cells. Y-axis shows the term description. Panels show up- and down-regulated gene sets. Shading is proportional to the p-value for the test and size to the proportion of annotated genes that contribute to the up- or down-regulation (i.e., ‘leading edge’ genes). Only terms with the top 30 enrichment scores for each time point are shown

Gene ontology (GO) term enrichment analyses were generally consistent across different time points post-inoculation, with many of the same terms occurring repeatedly among the most highly enriched gene sets (Fig. 7). Most of the strong enrichment scores were associated with down-regulated gene sets. There was a general decrease in expression of gene sets related to carbohydrate metabolism, ribosomal protein synthesis, and DNA replication, consistent with adaptations to a low-nutrient environment in the bee hindgut. There was also a decrease in expression of genes involved in responses to oxidative stress and detoxification (see Supplementary Data S4). These gene sets were largely overlapping, with leading-edge genes including catalases, glutathione and cytochrome peroxidases, and peroxiredoxins. Their upregulation in the culture environment could reflect responses to either oxidative or chemical stress. This could result from culturing under ambient oxygen levels as opposed to the relatively low oxygen levels in the gut [37]. Alternatively, it could reflect the fact that cultures were grown in antibiotic-enriched media, which was anecdotally reported to ‘massively impede’ growth of the related *C. bombi* and prevent establishment of new cell lines [54]. Upregulated gene sets were dominated by terms related to peptidase activity and amino acid metabolism his suggested a shift from carbohydrate- to amino acid-based substrates, similar to changes reported in other trypanosomatids in the insect gut vs. rich culture media [70] and conclusions from the initial publication describing this dataset [32].

We also found downregulation of gene sets related to microtubules, the cytoskeleton, and cell projections, with several flagellar proteins among the genes contributing to the downregulation (see Supplementary Data S4). This pattern was consistent with observations of reduced flagellum length and cell motility in attached haptomonad morphotypes carpeting the bee gut epithelium, as opposed to the freely swimming promastigotes found in log-phase cultures [22, 23]. This was countered by upregulation of gene sets related to protein and vesicle-mediated transport, macromolecule localization, and the endomembrane system (Supplementary Data S4), suggesting reorganization of cellular structure and intracellular machinery as cells proliferated along the gut wall (Fig. 7).

Our GO reanalysis differs from that of the original authors [32] in data inputs and methodological approach, but yields similar conclusions. Both analyses indicate increased expression of peptidases; but downregulation of genes involved in energy metabolism, protein synthesis, oxidative stress, and flagellar function. One gene set not noted by the original authors was the ABC transporters, which showed upregulation at the final time point (Supplementary Data S4). Leading edge genes included multiple paralogs of pentamidine resistance protein 1, ABC subfamily B and C proteins, and mitochondrial multidrug resistance proteins. This induction of expression could result from increased exposure to host-produced antimicrobial peptides and chemicals from plants and the environment as bees become foragers later in life [71]. Our analysis additionally identified upregulation of the cell adhesion gene set, with leading edge genes comprising five GP63/leishmanolysin paralogs, which we discuss further in the context of the KEGG analysis below.

#### KEGG enrichment suggests upregulation of glycan and amino acid synthesis, downregulation of flagellar genes in gut environment

**Fig. 8:**
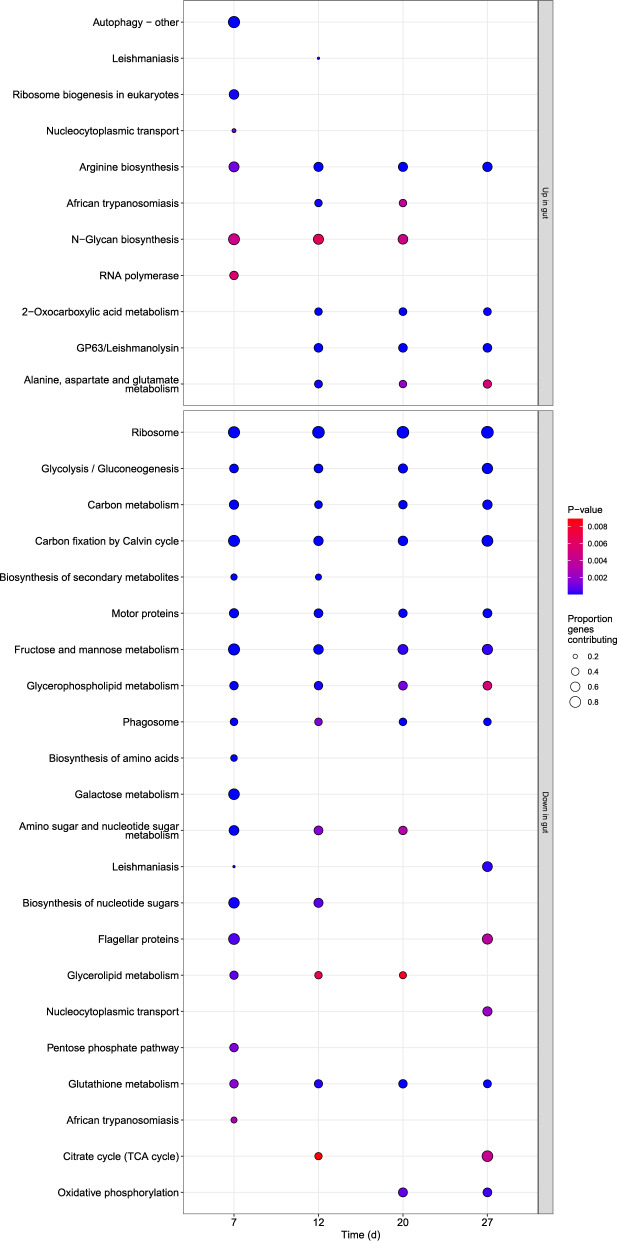
KEGG pathway gene set enrichment for *L. passim* expression levels in honey bee gut samples at 4 time points post-inoculation relative to cultured cells. Y-axis shows the term description. Panels show up- and down-regulated gene sets. Shading is proportional to the p-value for the test and size to the proportion of annotated genes that contribute to the up- or down-regulation (i.e., ‘leading edge’ genes)

Kyoto Encyclopedia of Genes and Genomes (KEGG) pathway analysis was concordant with the GO analysis, indicating downregulation of gene sets related to protein synthesis, energy metabolism and glutathione-based antioxidant metabolism, and flagellar and motor proteins. This was countered by upregulation of gene sets related to amino acid metabolism (arginine biosynthesis; 2-oxocarboxylic acid metabolism; and alanine, aspartate, and glutamate metabolism; Fig. 8). Upregulation of the autophagy-associated gene set at the first time point (7 d post-inoculation) was further suggestive of a nutrient-limited environment in the bee gut; this term was not highlighted by the original study’s GO term analysis [32]. We also found upregulation of the gene set encoding proteins for variants of GP63/leishmanolysin–a glycoprotein and peptidase involved in cell adhesion and cleavage of host effector proteins. The N-glycan biosynthesis-related gene set was likewise upregulated, which was not reported previously [32]. Upregulated genes in this set included several glycosyltransferases, which catalyze the attachment and removal of sugar and acetylglucosamine groups from proteins (Supplementary Fig. 8). Both GP63 and N-glycan biosynthesis gene sets are likely important for adherence to and interaction with host tissues or gut biofilms [72, 73].

Attachment to the insect gut epithelium is a process common to most of the trypanosomatids, with cell surface proteins playing a key role in the process [72]. In *C. fasciculata*, which like *L. passim* attaches to the hindgut wall and assumes a truncate morphology with a reduced flagellum, similar upregulation of GP63 proteins and genes involved in cell adhesion was observed during infection of mosquitoes as well as in adherent cultured cells [73]. Given that the attachment process generally occurs on the time scale of hours rather than days [72], closer attention in the period immediately post-inoculation could provide greater insight into how *L. passim* gene expression changes during initiation of infection.

Overall, the functional patterns of differential expression bear strong similarity to those reported in other trypanosomatids during both insect and mammal infection. Transcriptomes of *Herpetomonas muscarum* in *Drosophila* relative to cell cultures likewise indicated a pattern of increased amino acid utilization and autophagy [74]. Like the changes in *C. fasciculata* [73], they also showed reduced expression of flagellum-related genes and increased expression of multiple GP63 peptidases as well as other proteins associated with the cell surface [74]. In addition, post-inoculation upregulation of genes involved in DNA replication and repair in *H. muscarum* [74] was concordant with the upregulation of gene sets annotated for nucleic acid catalytic and helicase activities in our GO term enrichment analysis.

Beyond the insect gut, several aspects of *L. passim* gene expression in bees resembled those found in *Leishmania* species in the transition from the insect-associated promastigote form (in cell culture) to the amastigote form associated with vertebrate blood cells. These include down-regulation of gene sets associated with growth, carbon metabolism and flagellar motility; and upregulation of gene sets encoding peptidases and cell surface proteins [16, 75]. In light of these similarities, it is understandable how the same genes and related regions of the genome, such as the glycoprotein anchor and other surface protein-associated genes that are enriched on the ancestrally tetrasomic chromosome [14], could be important for both insect and vertebrate infection, and why this supernumerary region of the genome was enriched in genes upregulated during both *Leishmania* infection of blood cells [16] and *L. passim* infection of the bee gut described here. Maintaining and diversifying additional copies of such niche-specific, surface protein-encoding genes could enable parasites to continually evade recognition and subvert attack by current and novel hosts across evolutionary time.

#### Paralogous chromosomes contribute to upregulation of peptidase activity, amino acid synthesis, and ABC transporters

To explore the specific contributions of the paralogous Chromosomes 5 and 6 to differential gene expression in bees, we looked for gene sets upregulated during infection with at least 20% of their leading edge genes on Chromosomes 5 and 6 (i.e., more than expected given their combined ~ 12% of the genome size). There were 40 GO term-time point combinations that met these criteria. All but three of the terms involved some aspect of amino acid metabolism, including arginine and glutamine metabolic or biosynthetic processes and peptidase or carboxypeptidase activity at every time point; a consistent set of nine glutamyl carboxypeptidases on the paralogous chromosomes were among the leading edge genes. Eight KEGG pathway-timepoint combinations also met this criteria, of which seven included at least three leading-edge genes on the paralogous chromosomes. However, these comprised only two terms related to amino acid metabolism (2-oxocarboxylic acid metabolism and arginine biosynthesis); for each, the leading-edge genes matched the glutamyl carboxypeptidases highlighted by the GO analysis. These sets highlight the contributions of the paralogous chromosomes to the upregulation of amino acid metabolism, and specifically peptidase activity, that emerged from both GO and KEGG enrichment analyses.

The highest proportion of leading-edge genes on Chromosomes 5 and 6 (18/34, 53%) was found for the ABC-type (ATP-binding cassette) transporters upregulated at 27 d. These genes included four paralogs annotated as pentamidine resistance protein 1, four as putative mitochondrial p-glycoprotein or multidrug resistance protein E, six as ABC protein subfamily C, and two as subfamily D. These proteins have been implicated in drug resistance in *Leishmania* [76]. Their upregulation in the bee gut could enable parasite tolerance of the diverse phytochemicals found in nectar and pollen [77], which occur at concentrations sufficient to inhibit growth of *Leishmania* [78] and are proposed to have shaped the evolution of detoxification capacities in bees [79].

We also inspected the annotations of the remaining genes upregulated during infection (adjusted *P* < 0.05). Of 1,631 gene-timepoint combinations, only 23% were defined by the GO annotation and 12% by the KEGG; 74% had none. Peptidases were again prominent, including 34 cases of upregulation of glutamyl carboxypeptidases, eight of zinc metallopeptidases, and four of calpain family cysteine proteases. There were also 44 instances of upregulation of ABC-type transporters, 10 of zinc transporters, and 8 of carnitine transporters.

There were additional upregulated genes that could be important for attachment to the bee gut, remodeling of the cell surface, and interaction with host immune pathways. Putative modulators of cell adhesion included the calpains noted above – which play a role in *Trypanosoma cruzi* attachment to the tsetse fly midgut [80]–eight instances of upregulation of glycoprotease 2 genes, and three of GP63 genes. There were also five instances of upregulated phosphoglycan galactosyltransferases likely involved in lipophosphoglycan (LPG) synthesis and eight of integrin alpha chain protein. Upregulation of genes involved in vesicle-mediated transport–including endoplasmic reticulum vesicle transporters, BAR- and GOLD-domaining proteins, and Ras-like small GTP-asess – suggested an additional role of the paralogous chromosomes in transport of these mediators of attachment to the cell surface, while upregulation of genes involved in sterol synthesis (mevalonate kinase, sterol desaturase, and squalene synthase) could support remodeling of the cell membrane in the attached state. Also intriguing were six cases of upregulation of prostaglandin synthases, which could modulate host immune pathways. Collectively, these findings suggest that genes on the paralogous chromosomes modulate primary metabolism and nutrient acquisition, interactions with hosts, and tolerance of chemicals in the gut milieu.

#### Expression of leishmaniasis and trypanosomiasis infection-related pathway gene sets varies across time points

**Fig. 9:**
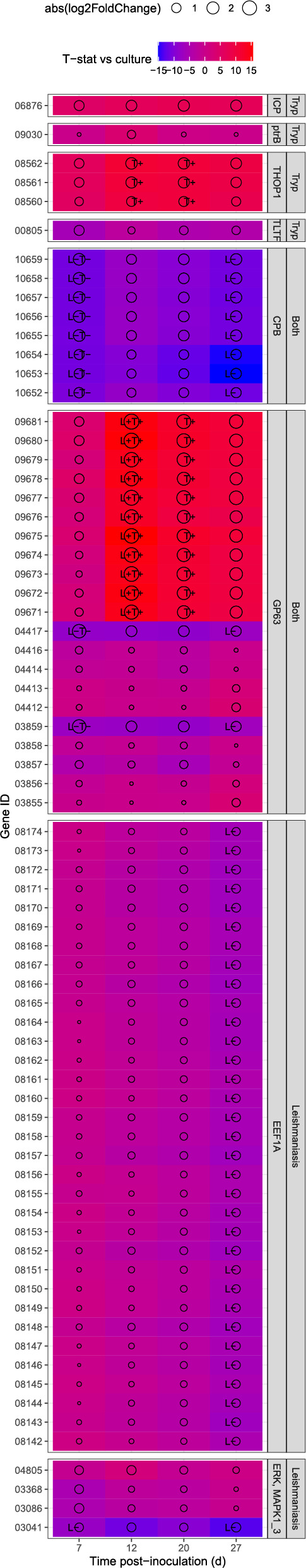
Differential expression of genes annotated as constituents of the ‘African trypanosomiasis’ (‘Tryp’) and ‘Leishmaniasis’ KEGG pathways. Shading corresponds to T-statistic for differential expression for bee gut vs. cultured cells at each time point post-inoculation. Open circles are sized in proportion to the log_2_-fold change. Y-axis numbers correspond to paralogs within each gene type, numbered consecutively according to their position in the genome. Panels labeled ‘both’ correspond to genes included in both the trypanosomiasis and leishmaniasis pathways. Letters and signs within the plot indicate genes most strongly contributing to up- (“+”) or downregulation (“-”) of the leishmaniasis (“L”) and trypanosomiasis (“T”) pathways at each time point. Abbreviations: ICP: inhibitor of cysteine peptidase; ptrB: protease (Oligopeptidase) B; THOP: thimet oligopeptidase (“Tropolysin”); TLTF: T lymphocyte-triggering factor; CPB: cysteine peptidase B; GP63: major surface glycoprotein 63 (“Leishmanolysin”); EEF1A: elongation factor 1-alpha; ERK: extracellular signal-regulated kinase, MAPK: mitogen-activated protein kinase

The most apparent fluctuations in differential expression across time points were found for gene sets annotated as components of the leishmaniasis and trypanosomiasis pathways, each of which appeared among the top up- or downregulated sets at different time points (Fig. 8). Both terms describe genes involved in trypanosomatid infection of mammals, consisting mostly of host immune pathways along with a few parasite-derived virulence factors and immunomodulators. The leishmaniasis pathway gene set was down-regulated at 7 d, upregulated at 12 d, then downregulated again at 20 d post-inoculation. This reflected strong downregulation of cysteine peptidase B (CPB) at 7 d and 20 d and downregulation of elongation factor 1-alpha (EEF1a) at 20 d, which was countered by strong upregulation of GP63-family genes from 12 d onwards (Fig. 9). The trypanosomiasis pathway gene set was downregulated at 7 d–again reflecting downregulation of cysteine peptidase B (CPB)–but upregulated at 12 and 20 d, reflecting increased expression of multiple paralogs of GP63 and thimet oligopeptidase (THOP1/tropolysin) (Fig. 9).

GP63 is noted for providing resistance to complement-mediated lysis in the bloodstream as well as to hydrolytic enzymes in the sand fly gut [81], whereas cysteine peptidase inhibits inflammatory signaling pathways [82]–including the JAK-STAT pathway important for clearing infection with the trypanosomatid *H. muscarum* in *Drosophila* [83]. Although both represent large protein families, this suggests that these genes could likewise protect parasites from host (bee) defenses or suppress their expression in response to infection. Indeed, very few changes in host gene expression occurred in *L. passim-*inoculated honey bees [32]; only modest and transient upregulation of immune genes occurred in honey bees inoculated with *C. mellificae* [84]; and most bumble bee immune genes showed little induction following inoculation with *C. bombi* [85], with the most infectious strains eliciting the weakest expression of antimicrobial peptides [86].

#### Uncertainty about expression of individual paralogs and abundance of protein products

Scrutiny of genes involved in the leishmaniasis and trypanosomiasis pathways reflected both the structure of the genome and the limitations of the RNAseq analysis. Strings of paralogs generally appeared as tandemly arrayed genes along the chromosome, including 2 sets of 5 paralogs of GP63 on the Chromosomes 5 and 6 and over 30 consecutive paralogs of EEF1A on Chromosome 18 (Fig. 9). Due to the sequence similarity among paralogs, sequencing reads could not be unambiguously assigned. These reads were distributed fractionally across each gene to which they aligned, resulting in read counts that were identical (or nearly so) among many spatially clustered paralogs, and non-independence of differential expression estimates for each one. This limitation in precision of mapping also likely inflated our estimates of correlations between transcript levels of neighboring genes (Fig. 6).

An additional limitation is our reliance on transcriptional data as the sole metric of gene expression. Abundance of mRNA correlates imperfectly with concentration of the corresponding proteins, and in *Leishmania* there is a dynamic relationship between the two [87]. Characterization of the proteome–recently described for *L. passim* in cell culture [88]–and its changes during different phases of infection would provide further insights into regulation of these ultimate gene products and how it compares with patterns of transcription.

## Conclusions

Our analysis leverages one of the first chromosome-level assemblies of an insect-specific trypanosomatid parasite of bees to illuminate the structural singularities of the genome and how they relate to gene expression, host infection, and parasite evolution. By linking the differentiation of an ancestrally tetrasomic region (i.e., Chromosome 31 of *L. major*) into two distinct, disomic chromosomes (i.e., Chromosomes 5 and 6 of *L. passim*) with the presence of genes upregulated in the honey bee host, our findings suggest a potential advantage of this salient genomic feature. Our transcriptomic analysis highlights shared patterns of gene expression across diverse trypanosomatids during host infection, as well as similarities between the functional changes that occur during infection of insects and vertebrates. The paralogous chromosomes, including genes for surface proteins that directly interface with hosts, appear to have disproportionate importance for establishment in both types of host. Hence, both elevated copy number and divergence of these chromosomes and their genes was likely advantageous in colonizing the diverse ecological niches occupied by trypanosomatids, including the hindgut of honey bees.

## Supplementary Information

Supplementary Data S1. RNAseq read counts used for transcriptome analysis.

Supplementary Data S2. GO assignments made by Pannzer2.

Supplementary Data S3. KEGG assignments made by BlastKoala.

Supplementary Data S4. GSEA results from the GO analysis.

Supplementary Data S5. GSEA results from the KEGG analysis.

Supplementary Information: Supplementary Tables, Figures, Methods, and description of data files.

## Data Availability

The *L. passim* BRL (2024) Hi-C and HiFi raw reads are available on NCBI GenBank (accession ID: SRX22798691 and SRX22798690); the assembled chromosomes (accession ID: GCA_037349495.1), maxicircle, and minicircles are listed under BioProject PRJNA1049372. The annotated genome is available on FigShare as a supplement to our previous article documenting the assembly [40] at https:/doi.org/10.25387/g3.27121227. RNA sequence data were used from NCBI BioProject PRJNA587465. The gene counts, GO terms assigned by Pannzer2, and KEGG terms assigned by BlastKOALA are provided as supplementary materials.
