# Supplementary material for: Chromosome-level genome assembly of trypanosomatid parasite Lotmaria passim links chromosome duplication and divergence with infection of honey bees
Source: BMC Genomics. 2025 Dec 1;26:1083. doi: 10.1186/s12864-025-12082-y (PMC12667155; doi:10.1186/s12864-025-12082-y)
Supplement: Supplementary file 6 — Supplementary Information: Supplementary Tables, Figures, Methods, and description of data files. [file 12864_2025_12082_MOESM6_ESM.docx]

Chromosome-level genome assembly of trypanosomatid parasite *Lotmaria passim* links chromosome duplication and divergence with infection of honey bees

Anthony Nearman^1✝^, Anzhelika Butenko^2,3,4,^ Jay D Evans^1*^, Evan C Palmer-Young^1*✝^

^1^USDA-ARS Bee Research Laboratory, 10300 Baltimore Ave, BARC-East Bldg. 306 Rm 313, Beltsville, MD 20705, USA

^2^Czech Academy of Sciences, Institute of Parasitology, České Budějovice 370 05, Czech Republic

^3^Life Science Research Centre, Faculty of Science, University of Ostrava, Ostrava 710 00, Czech Republic

^4^Faculty of Science, University of South Bohemia, České Budějovice 370 05, Czech Republic

^✝^These authors contributed equally.

*Corresponding authors:

JDE: [jay.evans@usda.gov](mailto:jay.evans@usda.gov)

ECPY: [evan.palmeryoung@gmail.com](mailto:evan.palmeryoung@gmail.com), [evan.palmer-young@usda.gov](mailto:evan.palmer-young@usda.gov)

# Supplementary Information

# Supplementary Tables

**Supplementary Table S1.** Genomes used for the synteny analysis. Strains are listed to disambiguate multiple assemblies on TriTrypDB.

| Species | Source |
| --- | --- |
| *Crithidia acanthocephali* TCC037E | NCBI (GCA_000482105.1) |
| *Crithidia bombi* 08.076 | NCBI (GCA_900240985.1) |
| *Crithidia expoeki* BJ08.175 | NCBI (GCA_900240875.1) |
| *Crithidia fasciculata* CfC1 | TriTrypDB-64 |
| *Leishmania braziliensis* MHOM/BR/75/M2904 | TriTrypDB-64 |
| *Leishmania major* Friedlin | TriTrypDB-64 |
| *Leptomonas pyrrhocoris* H10 | TriTrypDB-64 |

**Supplementary Table S2.** Genome assemblies used for the phylogenetic reconstruction.

| Species | Source |
| --- | --- |
| *Crithidia bombi* 08.076 | NCBI (GCA_900240985.1) |
| *Crithidia brevicula* S14 | NCBI (GCA_030849845.1) |
| *Crithidia expoeki* BJ08.175 | NCBI (GCA_900240875.1) |
| *Crithidia fasciculata* CfC1 | TriTrypDB-64 |
| *Crithidia mellificae* ATCC 30254 | NCBI (GCA_002216565.1) |
| *Crithidia thermophila* CT-IOC 054 | NCBI (GCA_030849055.1) |
| *Porcisia hertigi* LV43 | TriTrypDB-64 |
| *Leishmania braziliensis* MHOM/BR/75/M2904 | TriTrypDB-64 |
| *Leishmania donovani* HU3 | TriTrypDB-64 |
| *Leishmania mexicana* MHOM/GT/2001/U1103 | TriTrypDB-64 |
| *Leishmania major* Friedlin | TriTrypDB-64 |
| *Leptomonas pyrrhocoris* H10 | TriTrypDB-64 |
| *Leptomonas seymouri* ATCC 30220 | TriTrypDB-64 |
| *Lotmaria passim* SF | NCBI (GCA_000635995.1) |

**Supplementary Table S3.** Total counts of the most abundant tandem repeats between 5 and 15 bp, summed across the proximal and distal 1% of the length of the nuclear chromosomes.

| **Repeat sequence** | **Count** |
| --- | --- |
| AACCCTAACCCT | 9640 |
| AAACACACAC | 6414 |
| AACCCGTACACCCT | 5878 |
| AAAAAAG | 2778 |
| AACCCTAACCT | 1670 |
| AACCCCTAACCCT | 1509 |
| ACTCCTC | 1124 |
| ACACTACT | 1017 |
| AAACACAC | 973 |
| AACAC | 905 |
| AACCCT | 790 |
| ACGGGCTCG | 761 |
| ACACC | 580 |
| ACACCCGT | 560 |
| AAAAGG | 547 |
| ACACT | 425 |

**Supplementary Table S4.** Model summaries for the correlations between log2-fold changes of neighboring genes.

| Time | effect | group | term | estimate | std.error | statistic | p.value |
| --- | --- | --- | --- | --- | --- | --- | --- |
| 7 | fixed | NA | (Intercept) | -0.103 | 0.0186 | -5.52 | 3.39e-8 |
| 7 | fixed | NA | Neighbor | 0.24 | 0.0593 | 4.05 | 5.2e-5 |
| 7 | fixed | NA | Neighbor:Same.strand | -0.0232 | 0.0601 | -0.386 | 0.699 |
| 7 | random | Chrom | sd__(Intercept) | 0.0901 | NA | NA | NA |
| 7 | random | Residual | sd__Observation | 0.792 | NA | NA | NA |
| 12 | fixed | NA | (Intercept) | -0.0493 | 0.0129 | -3.81 | 1.4e-4 |
| 12 | fixed | NA | Neighbor | 0.268 | 0.0595 | 4.5 | 6.75e-6 |
| 12 | fixed | NA | Neighbor:Same.strand | -0.0173 | 0.0603 | -0.287 | 0.774 |
| 12 | random | Chrom | sd__(Intercept) | 0.0579 | NA | NA | NA |
| 12 | random | Residual | sd__Observation | 0.664 | NA | NA | NA |
| 20 | fixed | NA | (Intercept) | -0.0587 | 0.0125 | -4.68 | 2.87e-6 |
| 20 | fixed | NA | Neighbor | 0.327 | 0.0555 | 5.89 | 3.88e-9 |
| 20 | fixed | NA | Neighbor:Same.strand | -0.101 | 0.0563 | -1.79 | 0.0728 |
| 20 | random | Chrom | sd__(Intercept) | 0.055 | NA | NA | NA |
| 20 | random | Residual | sd__Observation | 0.658 | NA | NA | NA |
| 27 | fixed | NA | (Intercept) | -0.0221 | 0.0131 | -1.69 | 0.0905 |
| 27 | fixed | NA | Neighbor | 0.322 | 0.0538 | 5.99 | 2.07e-9 |
| 27 | fixed | NA | Neighbor:Same.strand | -0.0995 | 0.0546 | -1.82 | 0.0685 |
| 27 | random | Chrom | sd__(Intercept) | 0.0592 | NA | NA | NA |
| 27 | random | Residual | sd__Observation | 0.655 | NA | NA | NA |

Supplementary Figures


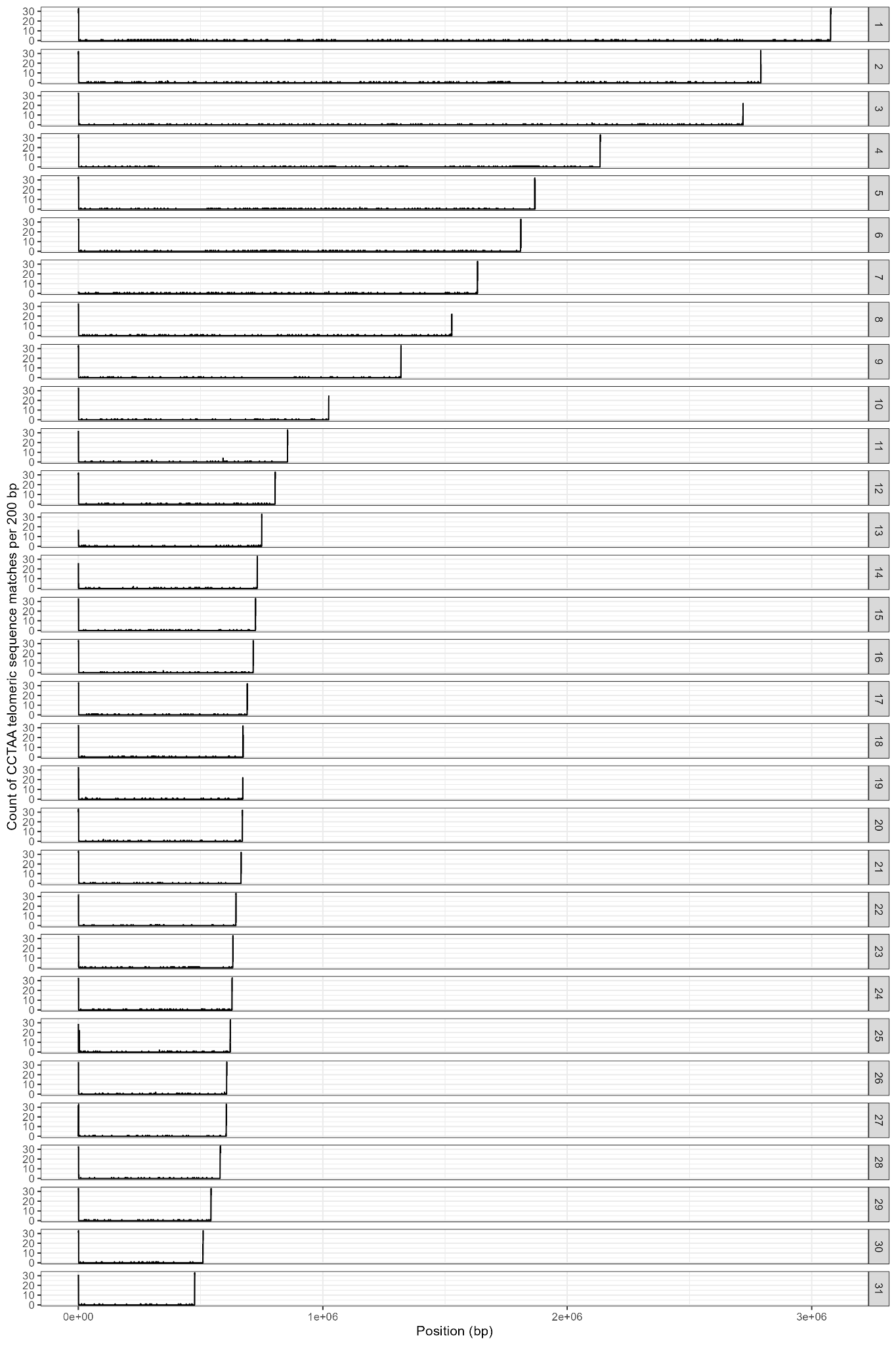


**Supplementary Figure 1. Number of matches to the sequence 'CCCTAA' and its reverse complement in each 200 bp sequence window of the assembled nuclear chromosomes.** X-axis shows position along the chromosome; y-axis shows number of matches; panels represent different chromosomes.

 
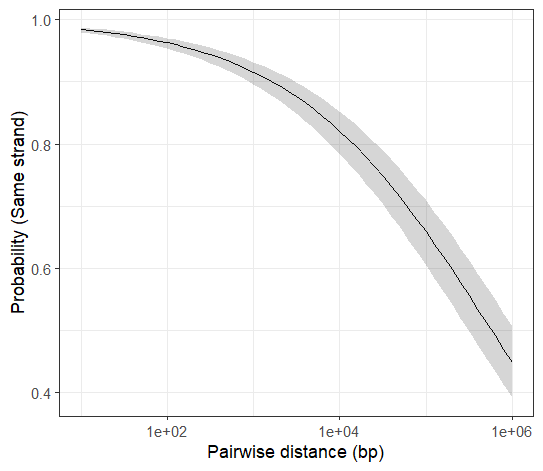


**Supplementary Figure 2. Relationship between pairwise distance between genes and probability that both are located on the same strand**. 97% of adjacent gene pairs are located on the same strand. Genes within 1 Kb of one another have a >90% chance of being on the same strand, and those within 10Kb have a >80% chance. Trendline and shaded band show estimated model means and 95% confidence intervals.


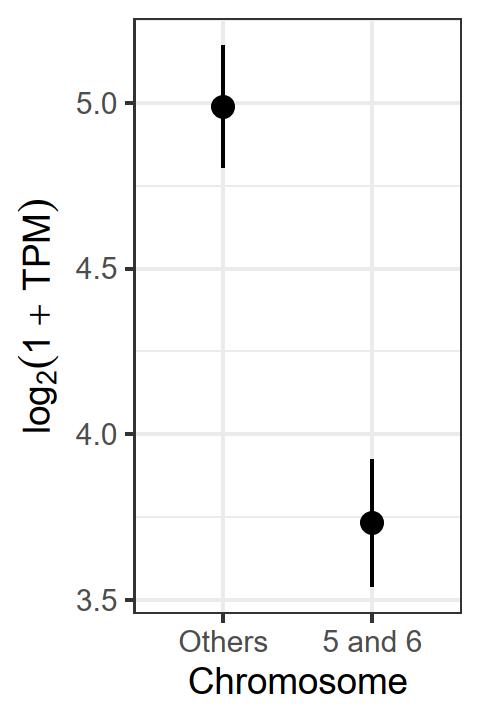


**Sup****plementary Figure 3. Paralogous Chromosomes 5 and 6** have lower per-gene expression levels relative to the rest of the genome. Points show model means and 95% confidence intervals. Y-axis shows log-transformed expression level in transcripts per million, pooled across all samples and excluding genes on the maxicircle.


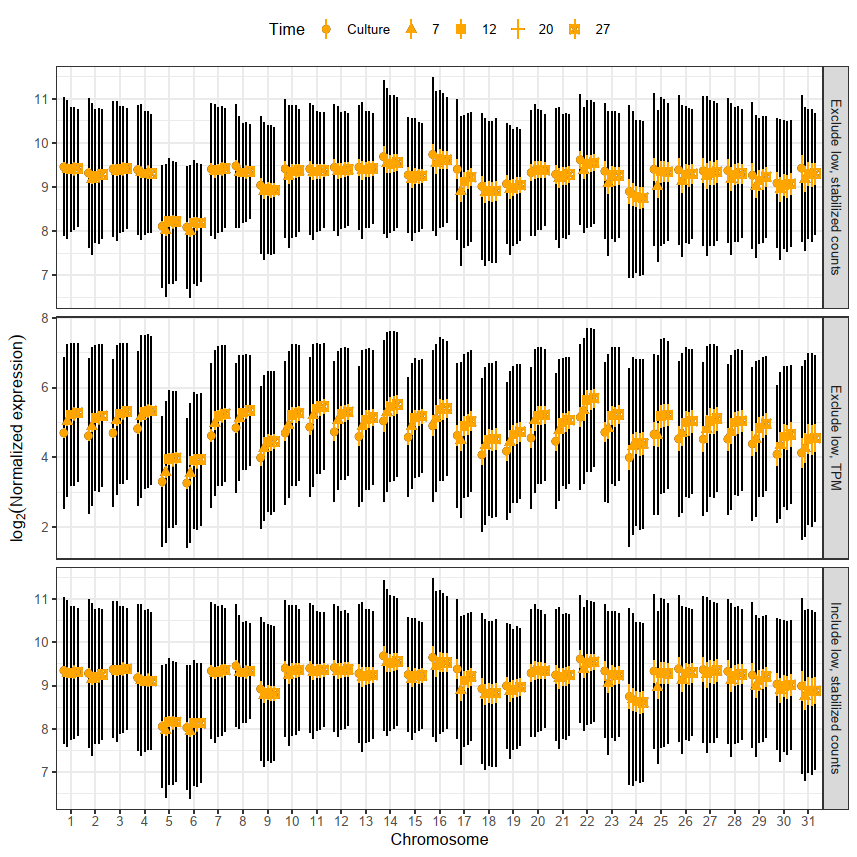


**Supplementary Figure 4. Paralogous Chromosomes 5 and 6 have lower per-gene expression levels** relative to the rest of the genome. Points show means ± 2 SE (orange) and ± 1 SD (black), with different shapes corresponding to each of the samples types (cultured cells and cells in bee guts at different time points post-inoculation). Y-axis shows transcripts per million or variance-stabilized read count, normalized to account for gene length, with or without exclusion of genes with low expression levels. “Maxi” refers to the mitochondrial maxicircle.

**
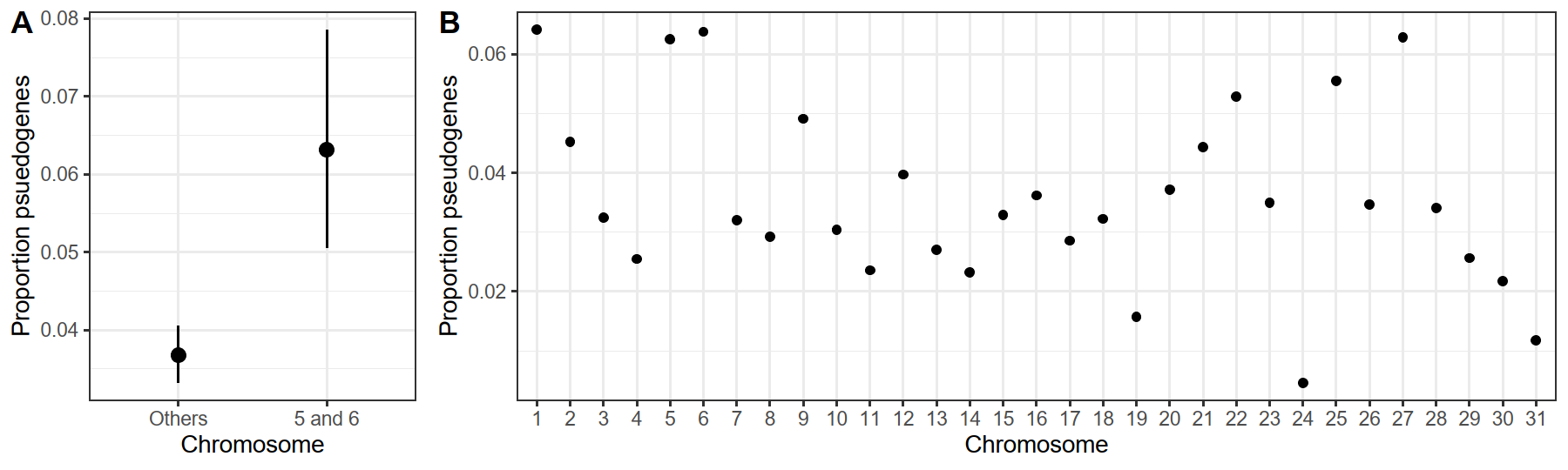
**

**Supplementary Figure 5. Paralogous Chromosomes 5 and 6 have a higher proportion of predicted pseudogenes** relative to the rest of the genome. (A) Points and error bars show model means and 95% confidence intervals from the binomial model. (B) Proportions of annotated genes on each chromosome that correspond to predicted pseudogenes.


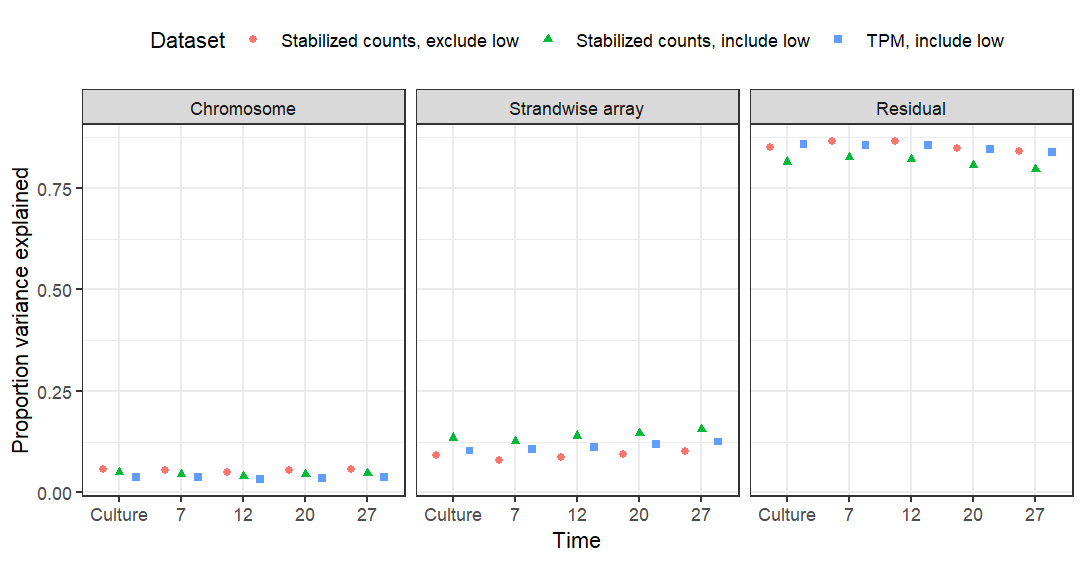


**Supplementary Figure 6. Same-stranded arrays explained 8-16% of variance in expression level** when modeled as a random effect. X-axis shows the five sample groups. Shapes indicate estimates for data sets with variance-stabilized read counts (with and without low-count genes) and transcripts per million (TPM) as the response variable.


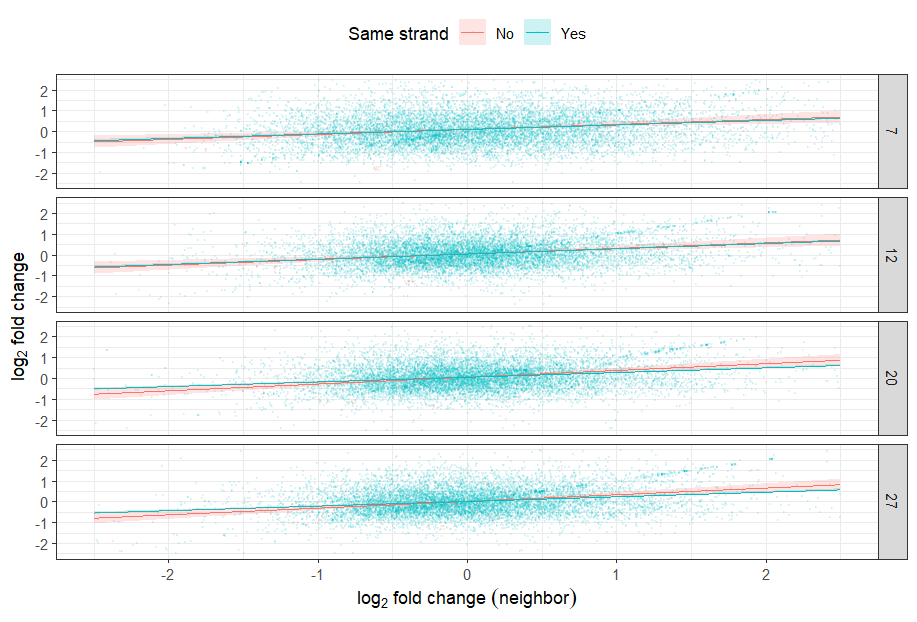


**Supplementary Figure 7. Adjacent genes are correlated in extent of differential expression.** Y- and X-axes show log_2_-fold changes of each pair of neighboring genes relative to cultured cells. Trendlines show correlations with 95% CI shaded bands, in red for neighbors on opposite strands and blue for neighbors on the same strand. Panels in rows indicate sample groups corresponding to different time points post-inoculation. For model summaries, see [Supplementary Table S4](#h3lssw67arz3).


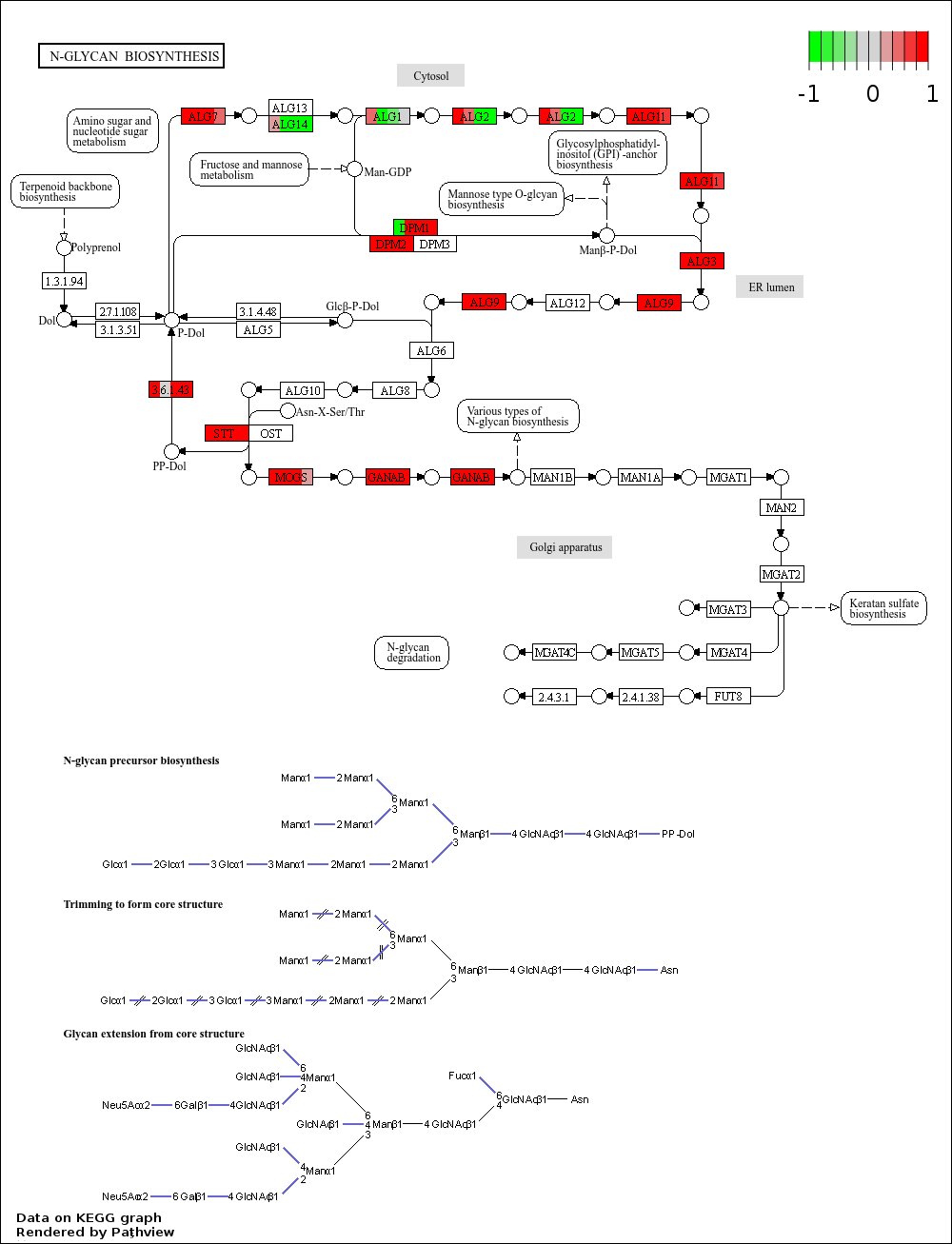
**Supplementary Figure 8. Differential expression of genes putatively involved in N-glycan biosynthesis.** Boxes denote genes or Enzyme Commission (EC) codes. Horizontally striped shading corresponds to the T-statistic for expression level in gut vs. culture at each time point post-inoculation (7, 12, 20, and 27 d). ALG: asparagine-linked glycosylation. DPM: dolichol-phosphate mannosyltransferase. GANAB: mannosyl-oligosaccharide alpha-1,3-glucosidase (neutral alpha-glucosidase AB). MOGS: mannosyl-oligosaccharide glucohydrolase. STT: dolichyl-diphosphooligosaccharide protein glycosyltransferase. The figure was rendered using the Pathview web server (pathview.uncc.edu).

# Supplementary Data

Supplementary Data S1. RNAseq read counts used for transcriptome analysis.

Supplementary Data S2. GO assignments made by Pannzer2.

Supplementary Data S3. KEGG assignments made by BlastKoala.

Supplementary Data S4. GSEA results from the GO analysis.

Supplementary Data S5. GSEA results from the KEGG analysis.

# Supplementary Methods

## Spatial analysis of genes and gene expression

Differential expression was analyzed using *DESeq2* [1] to compare expression in the bee gut relative to cell culture at each time point post-inoculation. Only genes covered by at least ten reads in at least five transcriptome libraries from each group were retained for further analysis, leaving 10100 genes of the original 10288 coding loci. Fractional counts were rounded to the nearest integer and normalized by library size (i.e., the total number of parasite-mapped counts). Differential expression in the bee gut relative to cell culture at each time point post-inoculation was assessed using negative binomial models with Benjamin-Hochberg correction for multiple testing within each time point. Variance-stabilized read counts, which approximate normalized read counts on the log_2_ scale and have similar variance throughout the range of counts, were computed based on dispersion parameter estimates for negative binomial distributed data and were used as the response variable for analysis of spatial clustering in expression [1].

The amount of variance in expression explained by same-stranded gene clusters of more than 10 consecutive genes was tested using linear mixed models. The response variable was the log_2_ scale variance stabilized read count, corrected for gene length by dividing each count by the length of the corresponding gene and multiplying by the median gene length for the whole data set. We ran separate models with and without the genes with low read counts that were excluded from differential expression analyses, and a third model with log_2_(transcripts per million (TPM)) as the response variable. In each case, chromosomes and gene clusters were used as random effects, and a separate model was fit for each of the 5 treatment groups. The proportion of total variance in read counts explained by the random effects was extracted using the ‘extract_variance_components’ function from R package *mixedup* [2].

Correlations in absolute and differential expression between adjacent genes was modeled with expression of each focal gene as the response variable; expression of the preceding gene, whether or not the two genes were on the same strand of the chromosome, and their interaction as predictor variables; and chromosome as a random effect. Separate models were fitted using variance-stabilized read counts– with and without low-count genes– and TPM as response variables for absolute expression (as for strand-wise gene clusters above) and log_2_-fold change vs cultured cells for differential expression. A separate model was fit for each treatment group (for absolute expression) or time point (for differential expression).

## Functional gene annotation and patterns of expression

Gene set enrichment analysis was implemented in *ClusterProfiler* [3]. The ranked gene list was used to calculate an enrichment score and associated p-value for the gene set associated with each GO term or KEGG pathway, along with identification of the ‘leading edge’ genes that contributed most strongly to each gene set’s pattern of differential expression [4]. The enrichment score reflects the extent to which the gene set is overrepresented at the top or bottom of the ranked list [4]. It is calculated as the maximum deviation from zero for a running-sum statistic. This is computed by proceeding down the full list of ranked genes, increasing the statistic when a gene belongs to the set and decreasing when it does not. The proportion of core enrichment or 'leading edge' genes reflect how many genes from the set have been encountered at the point when this maximum value of the running-sum statistic is reached, and hence contribute to the set's enrichment score. For the GO analysis, closely related terms with semantic similarity of 0.5 or higher were simplified to use only the term with the lowest p-value in each cluster of related terms [5].

## References

1. Love MI, Huber W, Anders S. Moderated estimation of fold change and dispersion for RNA-seq data with DESeq2. Genome Biol. 2014;15:550. https://doi.org/10.1186/s13059-014-0550-8.

2. Clark M. mixedup: Miscellaneous functions for mixed models. 2024.

3. Wu T, Hu E, Xu S, Chen M, Guo P, Dai Z, et al. clusterProfiler 4.0: A universal enrichment tool for interpreting omics data. The Innovation. 2021;2:100141. https://doi.org/10.1016/j.xinn.2021.100141.

4. Subramanian A, Tamayo P, Mootha VK, Mukherjee S, Ebert BL, Gillette MA, et al. Gene set enrichment analysis: A knowledge-based approach for interpreting genome-wide expression profiles. Proc Natl Acad Sci U S A. 2005;102:15545–50. https://doi.org/10.1073/pnas.0506580102.

5. Yu G, Li F, Qin Y, Bo X, Wu Y, Wang S. GOSemSim: an R package for measuring semantic similarity among GO terms and gene products. Bioinformatics. 2010;26:976–8. https://doi.org/10.1093/bioinformatics/btq064.
